# Dementia in Intellectual Disability: An Exploratory Investigation of Comorbidity Patterns and Diagnostic Outcomes

**DOI:** 10.1111/jir.70016

**Published:** 2025-08-20

**Authors:** Peer C. Keller, Tanja Sappok

**Affiliations:** ^1^ Bielefeld University, Medical School and University Medical Center OWL, Mara Hospital, University Clinic for People With Neurodevelopmental Disorders Bielefeld Germany

**Keywords:** Alzheimer's disease, challenging behaviour, dementia, Down syndrome, intellectual disability, neuroscience

## Abstract

**Background:**

Dementia is more prevalent and tends to manifest earlier in individuals with intellectual disabilities (ID) compared to the general population. Acquiring specific knowledge about comorbidities and diagnostic findings in individuals with ID who have dementia, as opposed to those with ID without dementia, is essential. Such insights are crucial for enhancing the quality of care.

**Methods:**

The study was applied in a German outpatient clinic for people with ID and mental illnesses from February 2018 to September 2022. An exploratory comparison was conducted to identify differences in somatic and psychiatric comorbidities, laboratory results, cerebrospinal fluid results, neuroimaging, medication and challenging behaviour in people with ID with (*n* = 13, mean age: 54 years, 69% female) and without dementia (*n* = 73, mean age: 53 years, 48% female).

**Results:**

In this sample, persons with ID who have dementia are more likely to have Down syndrome and less likely to have affective disorders. They received antidementia drugs more often and atypical high‐potency antipsychotics less often compared to persons with ID without dementia. All other clinical data showed no differences.

**Conclusions:**

Interestingly, no differences in somatic diseases (except Down syndrome) or laboratory and neuroimaging results could be found between people with ID with and without dementia. However, the diagnosis of dementia was associated with a reduced frequency of affective disorders and a reduced prescription of antipsychotics compared to the clinical sample without dementia. Due to the exploratory character of the study, replication in a much larger sample is necessary.

## 1 Background

As life expectancy rises among individuals with intellectual disabilities (ID), age‐related disorders like dementia are becoming increasingly prevalent (Dolan et al. 2019; Strydom et al. 2009). According to the World Health Organisation (2021, 2), ‘Dementia is a syndrome, usually of a chronic or progressive nature, caused by a variety of brain illnesses that affect memory, thinking, behaviour and ability to perform everyday activities’. ICD‐10 criteria‐defined dementia (regardless of type) is two to three times more common in the ID population (Strydom et al. 2009), although their life expectancy at birth is up to 20 years lower than for people without ID (Glover et al. 2017). This inevitably leads to a shift to younger age groups compared to the general population (Strydom et al. 2009). In people aged 60–69 years, the prevalence rate of dementia is about 2% in the general population versus about 9% in those with ID (Cao et al. 2020; Takenoshita et al. 2023). The predominant dementia subtype in people with ID is Alzheimer's disease, which accounts for about two thirds of dementia cases, roughly the same as in the general population (Strydom et al. 2007). A particularly high‐risk ID subgroup is individuals with Down syndrome, where the triplication of chromosome 21 leads to overexpression of the APP gene, a key factor in Alzheimer's disease pathogenesis (Fortea et al. 2021). Consequently, dementia occurs earlier and more frequently in this ID subgroup (Lautarescu et al. 2017); at least 50% of individuals with Down syndrome develop dementia by the age of 60 (Bayen et al. 2018).

The aim of this study is to compare a range of somatic, psychiatric and diagnostically relevant parameters in individuals with ID, both with and without dementia. A nuanced understanding of dementia within the ID population necessitates specialised knowledge to distinguish individuals with coexisting dementia from those without. These distinctions have profound implications for clinical practice, particularly in shared environments like ID residential care facilities, where tailored strategies are paramount.

Central to this understanding is recognising the distinct patterns of somatic and psychiatric comorbidities. Individuals with ID often contend with complex health profiles, including heightened risks of epilepsy, sensory impairments and chronic pain, alongside psychiatric conditions like depression or anxiety that may manifest atypically (Carvill 2001; El‐Tallawy et al. 2023; Hurley 2008; Sun et al. 2023). Clinicians must carefully disentangle these overlapping conditions to avoid misattributing symptoms solely to dementia. Comorbidities in dementia are frequently studied in the general population. A study by Schubert et al. (2006) with more than 3000 participants without ID from the United States found no variations in overall somatic comorbidity between patients diagnosed with dementia and those without. In a review that includes about 50 studies of the general population, the perspective is somewhat different; for example, diabetes and stroke seem to occur more frequently in people with dementia (Bunn et al. 2014). Recent reviews of studies in the non‐ID field highlight mental comorbidities, such as depression, which has been widely studied and has been shown to have associations with an increased risk of dementia (Stafford et al. 2022). However, findings may differ for people with ID due to atypical presentations of conditions like depression (Walton and Kerr 2016). Takenoshita et al. (2023) described various predictors for dementia in an ID sample, such as hypertension, depression, traumatic brain injury and stroke; vision disorder, hearing disorder and gait disorder were not associated with dementia in their study. Robertson et al. (2015) pointed out that for people with Down syndrome, epilepsy prevalence increases with age due to the increasing presence of dementia. However, this link is less clear for those with non–Down syndrome ID (van Schrojenstein Lantman‐de Valk et al. 1997). Meta‐analyses for the general population (Chen et al. 2024; Subota et al. 2017) have found positive associations between epilepsy and dementia; the prevalence rates depend on the different dementia subtypes. Both diseases affect each other, but causalities are difficult to assess. A key finding reported by Romoli et al. (2021) is that epilepsy probably accelerates cognitive decline.

Equally critical is the application of biomarkers, such as phosphorylated tau (a protein that accumulates in certain brain regions in dementia). Although biomarkers are valuable in general dementia diagnostics (Bjerke and Engelborghs 2018), their use requires adaptation for ID populations (Abdullah et al. 2025). Baseline cognitive differences, particularly in Down syndrome and severe or profound ID, may obscure traditional biomarker interpretations, necessitating specialised protocols to enhance diagnostic accuracy (Lee et al. 2017). So far, laboratory testing in dementia diagnostics is mostly used to preclude treatable causes of cognitive impairments. However, there have been many efforts to draw more conclusions from laboratory testing about dementia, as such tests are simple and inexpensive (Li et al. 2023). Blood‐based biomarkers and corresponding tests are relatively new and offer promising results (Leuzy et al. 2022). Further research is underway at cerebrospinal fluid (CSF) testing, which is used for the differential diagnosis of dementia (Llorens et al. 2016). According to Bjerke and Engelborghs (2018, 1199), there are ‘three core CSF biomarkers for Alzheimer's disease diagnostics, namely, the 42 amino acid long amyloid‐beta peptide [Aβ42], total tau protein, and tau phosphorylated [p‐tau]’. Neuroimaging, such as computed tomography (CT; cerebral computed tomography: cCT), magnetic resonance imaging (MRI; cerebral magnetic resonance imaging: cMRI) and electroencephalogram (EEG), is state of the art for the diagnosis of dementia (Klöppel et al. 2012). However, for the general population, Sullivan et al. (2012, 464) did ‘not suggest routinely screening patients with a clinical diagnosis of Alzheimer's disease using CT and MRI […]. The findings in CT and MRI are often seen in other conditions and are not necessarily specific to Alzheimer's disease’. For types of dementia other than Alzheimer's disease, such as vascular dementia, Sullivan et al. (2012) classified neuroimaging as indicated.

Medication management in ID emerges as another pivotal consideration, as polypharmacy risks are exacerbated by the frequent coexistence of chronic conditions (Sheerin et al. 2021). Clinicians must carefully differentiate dementia‐related cognitive decline from adverse drug effects, especially with anticholinergic medications, while optimising regimens to minimise interactions (Pieper et al. 2020). In a study group of over 50‐year‐olds with ID, almost 90% received at least one medication; polypharmacy was present in 39% (Peklar et al. 2017). At this high level of medication in the ID population, there may be differences in the intake of drugs between those people with ID with and without dementia. Axmon et al. (2017) showed that people with ID (and/or autism) who developed dementia were prescribed more antipsychotics and benzodiazepines than those without dementia. Confirming this pattern, Sheehan et al. (2015) reported a 42% higher antipsychotic prescription rate (incidence rate ratio: 1.42) in ID patients with dementia compared to all people with ID in their study.

Furthermore, addressing challenging behaviour demands a meticulous approach: dementia‐driven changes in aggression, apathy or withdrawal may closely resemble preexisting ID‐related behaviours (Hartley and MacLean 2007). Longitudinal monitoring is crucial to identify subtle deviations, guiding nonpharmacological interventions and reducing potentially inappropriate psychotropic treatments. Challenging behaviour is highly prevalent (approximately 18%) in people with ID (Bowring et al. 2017). Huxley et al. (2005) illustrated the differences in challenging behaviour between people with Down syndrome with or without dementia; the facets of lethargy and hyperactivity occurred more frequently in people with both Down syndrome and dementia. However, these associations prove difficult to study conclusively, as Lövheim et al. (2008) demonstrated that the relationship between cognitive impairment and challenging behaviour follows a nonlinear pattern, with peak prevalence typically occurring during midstage dementia. Although cross‐sectional studies with small samples cannot definitively establish these relationships, they can still reveal important trends, which remain the objective of this investigation.

By synthesising insights into the specific needs of individuals with ID and dementia, care providers can enhance diagnostic accuracy and develop individualised therapeutic interventions. This integration also enables the creation of supportive environments that effectively address the distinct requirements of ID subgroups, both with and without dementia. Such knowledge not only enhances individual care but also guides resource allocation and staff training in ID care settings, promoting a more responsive and equitable care system.

We hypothesise that individuals with ID who develop dementia will exhibit significant differences across multiple health and behavioural dimensions compared to those with ID who do not develop dementia. Although we make several assumptions, our investigation is primarily exploratory. Specifically, we expect to observe distinct comorbidity profiles between these groups, with the dementia cohort showing a higher prevalence and severity of age‐related and neurological conditions. Furthermore, differences in biomarker pathology are anticipated, including altered amyloid/tau ratios and inflammatory markers in laboratory and CSF results, as well as greater cortical atrophy, white matter lesions or hippocampal volume loss on cranial imaging (cCT and cMRI), and slower background rhythms or increased epileptiform activity on EEG. Medication regimens are also expected to differ, with the dementia group likely experiencing higher rates of polypharmacy and increased use of psychotropic and dementia‐specific medications. Lastly, we predict measurable contrasts in the nature, frequency and severity of challenging behaviour—such as greater agitation, aggression or apathy—among individuals with ID who develop dementia, suggesting an interaction between dementia progression and behavioural expression. Collectively, these anticipated differences aim to reveal nuanced disparities that could inform more tailored and effective clinical approaches for individuals with ID and dementia.

## 2 Materials and Methods

### 2.1 Study Design and Population

The present study analyses the medical data (details cf. below) of people with ID with and without dementia. Data were collected from February 2018 to September 2022 at a specialised outpatient clinic for mental health in people with ID in Berlin, Germany. This study is part of a larger research project that aimed to assess the psychometric properties of the Dementia Test for People with Intellectual Disability (DTIM; Rösner et al., unpublished manuscript; Sappok et al. 2025). The minimum age for inclusion was 55 years (40 years for individuals with Down syndrome because of the higher risk of early‐onset dementia, as described above), and the participants had to have a confirmed or suspected diagnosis of dementia. Profound intellectual disability, severe physical or sensory impairments, and lack of consent were exclusion criteria. Participants were recruited by personal contact in the medical consultation at the outpatient clinic and thus represent a clinical utilisation population. In total, 121 people with ID were recruited, with 35 dropouts before completion. The primary reasons for this dropout were limited cooperation (*n* = 14), the inability to conduct testing in a timely manner (*n* = 11) and the termination of treatment in the clinic (*n* = 5). In total, 86 people with ID completed the validation study; 13 (15%) were diagnosed with dementia. The decision in favour of a dementia diagnosis was made in a consensus conference based on the ICD‐10 criteria and all available clinical information, such as medical history, neurological and psychiatric examination, laboratory diagnostics and brain imaging, as well as neuropsychological testing and psychological questionnaires. All medical data were collected in accordance with good professional practice. For sample characteristics of the two ID subgroups with and without dementia, see Table 1.

**TABLE 1:** Sample characteristics.

	ID; no dementia	ID; dementia	Test statistic	*p*
Age	*M* = 52.74; SD = 11.76 (*n* = 73)	*M* = 54.46; SD = 6.39 (*n* = 13)	*U* = 453; *Z* = −0.260	0.795
Female *n*, %	35 of 73 (47.9%)	9 of 13 (69.2%)	OR = 2.443 (95%‐CI = 0.690–8.648)	0.230
Severity of ID				0.429
Mild *n*, %	16 of 73 (21.9%)	3 of 13 (23.1%)		
Moderate *n*, %	40 of 73 (54.8%)	9 of 13 (69.2%)		
Severe *n*, %	17 of 73 (23.3%)	1 of 13 (7.7%)		

*Note:* Mann–Whitney *U* test with *U* and *Z* values was reported for age. Exact Fisher's test, corresponding odds ratio and 95% confidence interval (CI) were reported for gender. Chi‐squared test was reported for severity of ID.

### 2.2 Medical Data

The collected clinical parameters were somatic and psychiatric comorbidities, laboratory results, CSF results, neuroimaging and medication. Most of them were dichotomous. Somatic and psychiatric comorbidities are as follows: present versus not present. Laboratory results, CSF results and neuroimaging are as follows: pathological or not pathological. Medication is taken versus is not taken (except for the number of medications, which is ratio scaled). The medical data, including medication, comorbidities, laboratory results, CSF results and neuroimaging data, were gathered from the current medical records in a conference with at least two but typically up to four involved doctors and psychologists. A specific data sheet for each patient was created. The accuracy and reliability of the data were ensured by the principle of multiple reviewers and final approval by the treating doctor. The investigation period was February 2018 to September 2022. The sample size varies depending on whether the parameters were collected as part of routine medical assessment or not. Only comorbidities and medication as clinical parameters were explicitly collected for the study and are therefore fully available for the sample. Given this, the results of laboratory, CSF and neuroimaging investigations are considered exploratory.

### 2.3 Challenging Behaviour

As a measure for challenging behaviour, the Aberrant Behaviour Checklist (Aman et al. 1985) in a German translation was filled out by people taking care of the participants prior to the study. It includes 58 items, such as ‘mood changes quickly’, and is answered on a 4‐point Likert scale from 0 (*the behaviour is not a problem*) to 3 (*the behaviour is a severe problem*) in the categories irritability (15 items), social withdrawal (16 items), stereotypic behaviour (7 items), hyperactivity/noncompliance (16 items) and inappropriate speech (4 items). Sums are calculated for the categories as well as an overall score. The scores can range between 0 and the number of items multiplied by 3 (overall max score = 174). Studies by the authors (Aman et al. 1987) and other researchers (Rojahn et al. 2011) confirm the reliability and validity of the Aberrant Behaviour Checklist.

### 2.4 Statistical Analysis

For the statistical analysis, IBM SPSS Statistics Version 29 was used. If values were missing, the cases were excluded from the analysis. All 2 × 2 crosstabs of dichotomous variables were compared using Exact Fisher's tests due to the small sample sizes. In addition, the odds ratio is reported if possible. A chi‐squared test was used for the severity of ID. To compare the number of medications and the Aberrant Behaviour Checklist with regard to the groups with and without dementia, Mann–Whitney *U* tests (two‐sided) were used. To avoid the probability of Type II errors (false negatives) becoming excessively high, the decision was made to forgo multiple testing correction. This trade‐off prioritises statistical power over strict control of Type I errors (false positives), which would have been reduced through methods like the Bonferroni adjustment.

## 3 Results

### 3.1 Somatic and Psychiatric Comorbidities

Participants with ID and dementia are more likely to have Down syndrome. No further differences in somatic comorbidities could be observed between participants with ID with and without dementia. With regard to psychiatric comorbidities, participants with dementia were less likely to have an affective disorder (cf. Table 2).

**TABLE 2:** Somatic and psychiatric comorbidities in participants with ID with and without dementia.

	ID; no dementia	ID; dementia	Test statistic	*p*
Hearing impairment *n*, %	5 of 73 (6.8%)	0 of 13 (0%)		1.00
Vision impairment *n*, %	8 of 73 (11%)	1 of 13 (7.7%)	OR = 0.677 (95% CI = 0.077–5.919)	1.00
Movement disorder *n*, %	6 of 73 (8.2%)	1 of 13 (7.7%)	OR = 0.931 (95% CI = 0.103–8.435)	1.00
Epilepsy *n*, %	10 of 73 (13.7%)	1 of 13 (7.7%)	OR = 0.525 (95% CI = 0.061–4.491)	1.00
Q90 (Down syndrome) *n*, %	25 of 73 (34.2%)	9 of 13 (69.2%)	OR = 4.320 (95% CI = 1.209–15.431)	**0.029**
Other somatic comorbidities *n*, %	34 of 65 (52.3%)	6 of 10 (60%)	OR = 1.368 (95% CI = 0.353–5.305)	0.742
F06 (other mental disorders due to brain damage […]) *n*, %	17 of 73 (23.3%)	5 of 13 (38.5%)	OR = 2.059 (95% CI = 0.594–7.130)	0.303
F1 (addictive disorders) *n*, %	4 of 73 (5.5%)	0 of 13 (0%)		1.00
F2 (schizophrenic disorders) *n*, %	17 of 73 (23.3%)	0 of 13 (0%)		0.063
F3 (affective disorders) *n*, %	42 of 73 (57.5%)	1 of 13 (7.7%)	OR = 0.062 (95% CI = 0.008–0.498)	**0.002**
F4 (anxiety, compulsive and trauma disorders) *n*, %	10 of 73 (13.7%)	0 of 13 (0%)		0.347
F8 (autism spectrum disorders) *n*, %	16 of 73 (21.9%)	0 of 13 (0%)		0.115

*Note:* Exact Fisher's tests, corresponding odd ratios and 95% confidence intervals (CI) were reported when calculable. Significant differences are marked in bold.

### 3.2 Laboratory Results

Laboratory tests did not differ between participants with ID with and without dementia. Details are given in Table 3.

**TABLE 3:** Pathological laboratory results in participants with ID with and without dementia.

	ID; no dementia	ID; dementia	Test statistic	*p*
Thyroid‐stimulating hormone (TSH) *n*, %	6 of 60 (10%)	2 of 12 (16.7%)	OR = 1.8 (95% CI = 0.317–10.222)	0.613
Triiodothyronine (T3) *n*, %	2 of 32 (6.3%)	0 of 10 (0%)		1.00
Thyroxine (T4) *n*, %	5 of 32 (15.6%)	3 of 10 (30%)	OR = 2.314 (95% CI = 0.442–12.114)	0.369
Thyroid peroxidase (TPO) *n*, %	5 of 10 (50%)	2 of 8 (25%)	OR = 0.333 (95% CI = 0.044–2.523)	0.367
Thyroglobulin antibodies (ATA) *n*, %	0 of 9 (0%)	1 of 8 (12.5%)		0.471
TSH receptor antibodies (TRAB) *n*, %	2 of 10 (20%)	1 of 8 (12.5%)	OR = 0.571 (95% CI = 0.042–7.740)	1.00
Vitamin B12 *n*, %	2 of 33 (6.1%)	1 of 12 (8.3%)	OR = 1.409 (95% CI = 0.116–17.116)	1.00
Folic acid *n*, %	9 of 32 (28.1%)	1 of 12 (8.3%)	OR = 0.232 (95% CI = 0.026–2.070)	0.241
Calcium *n*, %	0 of 24 (0%)	0 of 8 (0%)		1.00
Glycated haemoglobin (HbA1c) *n*, %	3 of 29 (10.3%)	0 of 10 (0%)		0.556
Lues serology *n*, %	0 of 14 (0%)	0 of 9 (0%)		1.00
Borrelia serology *n*, %	0 of 18 (0%)	1 of 9 (11.1%)		0.333

*Note:* Exact Fisher's tests, corresponding odds ratios and 95% confidence intervals (CI) were reported when calculable. Not every test was performed on every patient.

### 3.3 Cerebrospinal Fluid Results

The pathology of CSF results was equal in participants with ID with and without dementia (cf. Table 4).

**TABLE 4:** Pathological CSF results in participants with ID with and without dementia.

	ID; no dementia	ID; dementia	Test statistic	*p*
Cell count *n*, %	0 of 2 (0%)	0 of 4 (0%)		1.00
Protein *n*, %	0 of 2 (0%)	1 of 3 (33.3%)		1.00
Tau protein *n*, %	1 of 2 (50%)	2 of 3 (66.7%)	OR = 2.00 (95% CI = 0.051–78.250)	1.00
Phospho‐Tau protein *n*, %	1 of 2 (50%)	2 of 4 (50%)	OR = 1.00 (95% CI = 0.034–29.807)	1.00
β‐amyloid *n*, %	1 of 2 (50%)	2 of 4 (50%)	OR = 1.00 (95% CI = 0.034–29.807)	1.00
β‐amyloid ratio *n*, %	1 of 2 (50%)	1 of 1 (100%)		1.00

*Note:* Exact Fisher's tests, corresponding odds ratios and 95% confidence intervals (CI) were reported when calculable. Not every test was performed on every patient.

### 3.4 Neuroimaging

No differences could be observed in the pathology of neuroimaging cCT, cMRI and EEG between participants with ID with and without dementia, as can be seen in Table 5.

**TABLE 5:** Pathological neuroimaging in participants with ID with and without dementia.

	ID; no dementia	ID; dementia	Test statistic	*p*
cCT *n*, %	6 of 15 (40%)	2 of 4 (50%)	OR = 1.50 (95% CI = 0.164–13.749)	1.00
cMRI *n*, %	7 of 12 (58.3%)	4 of 5 (80%)	OR = 2.857 (95% CI = 0.241–33.902)	0.600
EEG *n*, %	10 of 14 (71.4%)	2 of 3 (66.7%)	OR = 0.800 (95% CI = 0.056–11.504)	1.00

*Note:* Exact Fisher's tests, corresponding odds ratios and 95% confidence intervals (CI) were reported. Not every test was performed on every patient.

### 3.5 Medication

Participants with ID with dementia received antidementia drugs more often and atypical high‐potency antipsychotics less often in comparison to participants with ID without dementia, as shown in Table 6. There was no difference regarding other psychotropic drugs, neither in the number nor in the type of drug.

**TABLE 6:** Medication in participants with ID with and without dementia.

	ID; no dementia	ID; dementia	Test statistic	*p*
Medication at all *n*, %	64 of 73 (87.7%)	10 of 13 (76.9%)	OR = 0.469 (95% CI = 0.108–2.032)	0.380
Classic high‐potency antipsychotics *n*, %	5 of 73 (6.8%)	0 of 12 (0%)		1.00
Atypical high‐potency antipsychotics *n*, %	29 of 73 (39.7%)	1 of 12 (8.3%)	OR = 0.138 (95% CI = 0.017–1.126)	**0.049**
Low‐potency or medium‐potency antipsychotics *n*, %	12 of 73 (16.4%)	2 of 12 (16.7%)	OR = 1.017 (95% CI = 0.197–5.238)	1.00
Antidepressants *n*, %	38 of 73 (52.1%)	4 of 12 (33.3%)	OR = 0.461 (95% CI = 0.127–1.665)	0.351
Anticonvulsants *n*, %	15 of 73 (20.5%)	1 of 12 (8.3%)	OR = 0.352 (95% CI = 0.042–2.941)	0.449
Lithium *n*, %	2 of 73 (2.7%)	0 of 12 (0%)		1.00
Benzodiazepine *n*, %	1 of 73 (1.4%)	0 of 12 (0%)		1.00
Methylphenidate *n*, %	0 of 73 (0%)	0 of 12 (0%)		1.00
Antidementia *n*, %	0 of 73 (0%)	7 of 12 (58.3%)		**< 0.001**
Antidiabetic *n*, %	5 of 73 (6.8%)	1 of 12 (8.3%)	OR = 1.236 (95% CI = 0.132–11.608)	1.00
Antihypertensive *n*, %	18 of 73 (24.7%)	2 of 12 (16.7%)	OR = 0.611 (95% CI = 0.122–3.054)	0.723
Thyroid medication *n*, %	16 of 73 (21.9%)	5 of 12 (41.7%)	OR = 2.545 (95% CI = 0.711–9.103)	0.160
Number of medications	*M* = 2.22; SD = 1.45 (*n* = 72)	*M* = 2.00; SD = 1.91 (*n* = 12)	*U* = 362; *Z* = −0.915	0.360

*Note:* Exact Fisher's tests, corresponding odds ratios and 95% confidence intervals (CI) were reported when calculable. For the number of medications, the Mann–Whitney *U* test with *U* and *Z* values was reported. Significant differences are marked in bold.

### 3.6 Challenging Behaviour

The mean values in the total test results and the different subscales of the aberrant behaviour checklist show no differences for participants with ID with dementia and without dementia (cf. Table 7).

**TABLE 7:** Means in the Aberrant Behaviour Checklist in participants with ID with and without dementia.

	ID; no dementia (*n* = 66)	ID; dementia (*n* = 9)	Test statistic	*p*
Irritability	*M* = 9.33; SD = 9.18	*M* = 7.00; SD = 6.91	*U* = 257.5; *Z* = −0.646	0.518
Social withdrawal	*M* = 7.89; SD = 7.09	*M* = 6.33; SD = 7.18	*U* = 244.5; *Z* = −0.860	0.390
Stereotypic behaviour	*M* = 2.85; SD = 3.72	*M* = 1.22; SD = 1.3	*U* = 240.5; *Z* = −0.958	0.338
Hyperactivity/noncompliance	*M* = 7.35; SD = 7.82	*M* = 2.56; SD = 2.51	*U* = 185.0; *Z* = −1.834	0.067
Inappropriate speech	*M* = 3.00; SD = 3.18	*M* = 3.11; SD = 2.62	*U* = 263.5; *Z* = −0.555	0.579
Sum score	*M* = 30.27; SD = 23.55	*M* = 20.22; SD = 17.8	*U* = 220.0; *Z* = −1.256	0.209

*Note:* Mann–Whitney *U* tests with *U* and *Z* values were reported.

## 4 Discussion

The aim of the study was to compare a range of somatic, psychiatric and diagnostically relevant parameters in individuals with ID, both with and without dementia. In this sample, persons with ID with dementia were more likely to have Down syndrome and less likely to have affective disorders. They received antidementia drugs more often and atypical high‐potency antipsychotics less often compared to persons with ID without dementia. All other clinical data showed no statistically significant differences.

In our sample, affective disorders occurred less frequently in the group with ID with dementia compared to those with ID without dementia. In fact, affective disorders are known risk factors for the development of dementia (Da Silva et al. 2013; Takenoshita et al. 2023). Several factors may explain this apparent contradiction. Da Silva et al. (2013) reviewed longitudinal studies and found that observations of disease progression are helpful for precise differentiation, especially in cases of diseases like depression and dementia. The current analysis was only a cross‐sectional study in a clinical sample. Here, affective disorders may mimic dementia because memory impairments could be attributed to depression. Therefore, it may be an important differential diagnosis of dementia—just like in people without ID as well (‘pseudodementia’, cf. Kang et al. 2014).

The higher prevalence of Down syndrome in the group of persons with ID and dementia was expected (Huxley et al. 2005), but this creates methodological challenges, as it essentially produces two distinct ID subgroups that could confound comparisons. Due to the limited sample size, these subgroups could not be analysed separately. In clinical practice, dementia should routinely be included in the differential diagnosis for individuals with Down syndrome, even at younger ages. This retrospective clinical study showed no further difference in somatic comorbidities between people with ID with and without dementia. This aligns with the findings of Schubert et al. (2006) for the general population and the findings of Takenoshita et al. (2023) for the ID population. Specifically, sensory impairments, movement disorders and epilepsy showed no association with dementia status in our clinical sample.

Additionally, the biomarkers, including laboratory results, CSF results and neuroimaging, revealed no differences between those with ID with and without dementia. The limited number of test results available for each group is a major limitation. Therefore, the study is exploratory, and further replication in a much larger sample is necessary. Should these null findings persist in larger studies, several biological factors unique to ID populations could explain them. Individuals with ID display considerable biological diversity, driven by a wide range of genetic and etiological factors that contribute to their cognitive impairments (Granholm and Ledreux 2021). This may alter biomarker expression or interpretation. Also, the baseline functioning of individuals with ID may inherently meet the criteria for pathology (Elliott‐King et al. 2016). Current clinical practice appropriately mirrors general population standards, with routine exclusion of treatable causes of cognitive decline. Nevertheless, targeted research accounting for ID‐specific characteristics remains imperative.

In our clinical sample, the mean number of medications was two in both study groups. The high medication usage among people with ID in this clinical sample is in line with other studies (Peklar et al. 2017; Sheerin et al. 2021). There were few differences in medication between individuals with ID with and without dementia. As could be expected, antidementia drugs were prescribed more frequently to people with ID and dementia. Contradicting the results of Axmon et al. (2017) and Sheehan et al. (2015), atypical high‐potency antipsychotics were given more often to those with ID without dementia. The clinical control group may have caused this unexpected finding—all controls were patients having psychiatric disorders. This is also reflected by the higher prevalence rates of schizophrenia in the nondementia ID group (23.3% vs. 0%), though these differences did not reach statistical significance. When prescribing specific medications for dementia in individuals with ID, it is essential to consider their high overall medication use and the potential for drug interactions.

Notably, no group differences could be observed for the severity of challenging behaviour between the groups with ID with and without dementia. This may also be due to the clinical sample of people with ID and mental disorders. There could be a general accumulation of challenging behaviour in both groups. Importantly for treatment and support, potential associations between dementia and challenging behaviour in ID remain unclear. The dementia diagnostic process must be based on dementia‐specific diagnostic instruments. In our sample, the consideration of challenging behaviour would not show diagnostic utility. These results highlight the need to investigate broader behavioural dimensions beyond traditional challenging behaviour, as more subtle behavioural changes might prove more informative for dementia identification in ID populations. Future research should therefore explore a wider spectrum of problematic behaviours while accounting for the confounding effects of psychiatric comorbidities prevalent in clinical samples.

### 4.1 Limitations

Interpretation of the findings requires caution due to the small sample size and limited clinical data available. Although the results showed no differences, this does not definitively rule out the existence of actual group differences; the study may have been underpowered to detect them. We deliberately abstained from applying Bonferroni correction to avoid exacerbating the risk of Type II errors (false negatives), though this decision simultaneously increases the possibility of Type I errors (false positives) among any observed effects. Further important limitations must be acknowledged. First, individuals with Down syndrome likely represent a distinct subgroup that should be analysed separately from those with ID due to other causes. Second, our clinical sample—drawn from healthcare system users—may limit generalisability due to treatment‐seeking biases. Future research would benefit from more controlled sampling strategies (particularly regarding comorbid mental illnesses), consideration of additional confounding variables and larger sample sizes to improve statistical power.

## 5 Conclusion

This study offers initial insights into potential diagnostic differences between people with ID with and without dementia. It must be noted that only limited findings could emerge due to the study's considerable limitations. We recommend further research on the differences between people with ID with and without dementia. Particular attention should go to accurate study planning, an adequate sample (consideration of covariates such as age, gender, severity of ID and Down syndrome) and longitudinal studies. A more in‐depth exploration of the findings from this study would be especially valuable to enable coordinated care across various settings, such as clinics or residential facilities for individuals with intellectual disabilities.

## Ethics Statement

The present study was conducted in full compliance with the ethical guidelines established by the relevant national and institutional committees for research involving human participants, as outlined in the Declaration of Helsinki (1975, revised in 2008). The ethics committee of Königin Elisabeth Herzberge Hospital in Berlin granted ethical approval for the study on 5 April 2017. Additionally, approval was obtained from the ethics committee of Ostfalia University of Applied Sciences in Wolfenbüttel on 28 June 2017 (Resolution Number 2/2017). Written informed consent was secured from all participants or their legal representatives prior to their involvement in the study. To ensure accessibility, both the consent forms and study information were provided in standard German as well as simplified German versions designed for ease of understanding.

## Conflicts of Interest

The authors declare no conflicts of interest.

## Data Availability

The data that support the findings of this study are available on request from the corresponding author. The data are not publicly available due to privacy or ethical restrictions.
